# Bacterial aggregate size determines phagocytosis efficiency of polymorphonuclear leukocytes

**DOI:** 10.1007/s00430-020-00691-1

**Published:** 2020-09-02

**Authors:** Maria Alhede, Melanie Lorenz, Blaine Gabriel Fritz, Peter Østrup Jensen, Hans Christian Ring, Lene Bay, Thomas Bjarnsholt

**Affiliations:** 1 Costerton Biofilm Center, Department of Immunology and Microbiology, University of Copenhagen, Blegdamsvej 3B, 2200 Copenhagen, Denmark; 2 Department of Clinical Microbiology, Rigshospitalet, Afsnit 9301, Juliane Maries Vej 22, DK-2100 Copenhagen Ø, Denmark; 3 Institute for Inflammation Research, Center for Rheumatology and Spine Diseases, Rigshospitalet, 2100 Copenhagen, Denmark; 4 Department of Dermatology, Bispebjerg Hospital, Nielsine Nielsens Vej 9, København, NV Denmark

**Keywords:** *E. coli*, *P. aeruginosa*, *S. aureus*, *S. epidermidis*, Chronic bacterial infection, Polymorphonuclear leukocytes (PMNs), Phagocytosis

## Abstract

The ability of bacteria to aggregate and form biofilms impairs phagocytosis by polymorphonuclear leukocytes (PMNs). The aim of this study was to examine if the size of aggregates is critical for successful phagocytosis and how bacterial biofilms evade phagocytosis. We investigated the live interaction between PMNs and *Pseudomonas aeruginosa*, *Staphylococcus aureus*, *Escherichia coli* and *Staphylococcus epidermidis* using confocal scanning laser microscopy. Aggregate size significantly affected phagocytosis outcome and larger aggregates were less likely to be phagocytized. Aggregates of *S. epidermidis* were also less likely to be phagocytized than equally-sized aggregates of the other three species. We found that only aggregates of approx. 5 μm diameter or smaller were consistently phagocytosed. We demonstrate that planktonic and aggregated cells of all four species significantly reduced the viability of PMNs after 4 h of incubation. Our results indicate that larger bacterial aggregates are less likely to be phagocytosed by PMNs and we propose that, if the aggregates become too large, circulating PMNs may not be able to phagocytose them quickly enough, which may lead to chronic infection.

## Introduction

Polymorphonuclear leukocytes (PMNs) are part of the innate immune system and constitute the first line of host defense against infectious pathogens. PMNs are phagocytes and utilize phagocytosis to engulf and destroy microorganisms. Phagocytosis is an active, receptor-mediated process by which bacteria are recognized, bound by the PMN's cell membrane and internalized into a phagosome. Granules containing antimicrobial molecules then mobilize, fuse with the phagosome and kill the bacteria [1, 2]. PMNs can expand up to 300% during phagocytosis, allowing the uptake of large particles [3]. Failure of phagocytosis may result in the formation of neutrophil extracellular traps (NETs) with pro-inflammatory effect [4].

Pathogenic bacteria have developed a variety of efficient strategies to avoid phagocytosis, engulfment and degradation by PMNs. One such strategy is the establishment of aggregates, also known as biofilms, which increase bacterial tolerance to challenges from the immune system and/or antibiotic treatment. Acute infections are assumed to involve planktonic bacteria and are generally treatable with antibiotics [5]. In cases where bacteria succeed in forming biofilms within the host, clinically intractable chronic infections may occur [6, 7]. The development of chronic infections may be partially attributed to the lack of successful phagocytosis by PMNs [8]. Biofilms are found in many infections [9], such as chronic wounds [10], catheters and in the lungs of cystic fibrosis (CF) patients [9].

The majority of research examining the interplay between PMNs and bacteria has focused on single bacterial cells [11]. Thus, the literature on interactions between bacterial aggregates and PMNs is limited. Bacteria residing in the human skin and oral microbiome are present as small aggregates [12]. These bacteria can be introduced into the body during tooth brushing, dental work, skin trauma and surgery, but are often quickly phagocytosed and eradicated. In a few cases, e.g. inserted implants, bacteria may cause either acute or chronic infections. In general, chronic infections are observed in 1–2% of patients with implants [13, 14]. It is not known if bacteria enter the body as single cells or aggregates during the transition from being part of the healthy microbiome to an infectious organism. Interestingly, it has previously been suggested that the size of invading aggregates may restrict phagocytosis by the PMNs. The authors proposed that phagocytosis by PMNs might fail if bacterial aggregates become too large [15].

In this study, we investigated whether a size restriction exists for the successful phagocytosis of aggregated bacteria by PMNs. The size of an aggregate may influence a PMN’s ability to eradicate it, thus initiating an infection. Phagocytotic efficiency and killing of PMNs were investigated for four bacterial species capable of causing chronic infection: *Pseudomonas aeruginosa*, *Staphylococcus aureus*, *Escherichia coli* and *Staphylococcus epidermidis.* Bacteria were grown to obtain both single cells and aggregated cells of various sizes before being combined with isolated human PMNs in vitro. Planktonic and aggregated bacteria of the four strains were compared by confocal scanning laser microscopy (CSLM) to visualize the phagocytosis of bacteria and the killing of PMNs. We additionally estimated the aggregate size of the bacteria residing on healthy skin to evaluate the relevance of our finding. This may provide an explanation for how bacterial biofilms establish infection within the human body, which is critical knowledge for successful prevention and treatment.

## Materials and methods

### Bacterial strains

*P. aeruginosa* (PAO1) obtained from the Pseudomonas Genetic Stock Center (www.ecu.edu/pseudomonas; strain PAO0001), *S. epidermidis* from a nose (ATCC 14990;(HUGH und ELLIS 1968)), a *E. coli* from a child with diarrhoea (042) [16] and *S. aureus* strain NCTC 8325 named *S. aureus* 8325–4 were used [17]. All strains are displayed in Table 4.

For *P. aeruginosa* rhamnolipid has been shown to be an important factor for killing of PMNs and can induce lysis of PMNs [18]. Therefore, we used a *P. aeruginosa rhlA* mutant (PA-*rhlA*), which is unable to produce rhamnolipid. For *S. aureus* we used a *spA* mutant (SA-*spA*) which will allow PMNs to phagocytose the bacteria. However, this mutant is still able to kill the PMNs from within due to the production of phenol soluble modulins (PSMs) that induce lysis [19].

### Growth conditions

Frozen bacterial stocks ( – 80° C) were streaked for isolation on Luria–Bertani (LB) agar plates and incubated overnight at 37 °C. For planktonic bacteria, an isolated colony was inoculated into 5 ml LB or 5 ml tryptic soy broth (TSB) in 15 mL culture tubes (sterile culture tubes, 6700090, Th. Geyer) and incubated for 20–22 h at 37 °C with shaking 180 rpm. The tubes were allowed to stand on the benchtop for 10 min to let aggregates settle. The top (1 ml) of the tube was transferred into 1.5 mL microcentrifuge tube. OD was measured at 450 nm and the sample was diluted to OD 0.1 in Krebs–Ringer BSS [20].

To generate aggregates of the bacteria, 10 ml LB (PA and EC) or TBS (SA, SE) in a 50 ml Erlenmeyer flasks was inoculated with an isolated colony (see above) and incubated at 37 °C for 20–22 h at 180 rpm. The flasks were allowed to stand on the table 10 min to let aggregates settle to towards the bottom leaving the single cells at the top before proceeding. One milliliter from the bottom of the flask was transferred to a 1.5 ml microcentrifuge tube. OD of the aggregates was measured at 450 nm and diluted to OD 0.1 in Krebs–Ringer BSS. For PA-*rhlA* mutant the aggregates were grown in 5 ml tubes and 1 ml was taken from the bottom of the tube.

### Isolation of PMN

Isolation of PMNs was done as described previously in [21]. In short twenty ml blood was drawn into EDTA tubes (BD Vacutainers K2E (EDTA), REF 367525). Twenty ml of blood was carefully layered on top of 20 ml PolymorphPrep (1114683, Alere, Denmark) in a 50 ml centrifuge tube. The blood was centrifuged for 25 min in a swing-out rotor (20 °C) without softstop or softstart at 500 g and 1 min before finishing the speed (g) was increased to 700 g for an additional 10 min. The lower band (PMNs) was harvested into a 50 mL centrifuge tube with a Pasteur glass pipette. Approximately 5 ml of PMNs were harvested per run. Cells were washed by filling the centrifuge tube with Hanks’ balanced salt solution (HBSS, H6648, Sigma) without Ca^2+^ and Mg^2+^ and with NaHCO_3_ (room temp.) to the 40 ml mark and centrifuged at 400 g for 10 min (20 °C). Additional red blood cells were removed using 10 mL ACK lysing buffer (Gibco, ThermoFisher scientific, A1049201) for 3 min followed by centrifugation for 5 min at 300 g (20 °C). The pellet was resuspended in 10 ml HBSS without Ca^2+^ and Mg^2+^ (RT) and centrifuged for 10 min at 120 g. Cells were resuspended in 10 ml HBSS without Ca^2+^ and Mg^2+^ at room temperature and stored at room temperature while counting cells. The concentration of PMNs was determined either by manual counting or using a NucleoCounter NC200 (Chemometec). PMNs were diluted to 4 × 10^6^ PMNs/ml in Krebs–Ringer BSS to a final concentration of 1 × 10^6^ PMNs/ml when mixed with bacteria in the microtiter plate. For experiments with SYTO9 ( 5 mM, S34934, ThermoFisher) and PI (0.2 μl/ml of 20 mM in DMSO, Sigma P4170-500 mg), SYTO9 was diluted 1000× (stock 5 mM in DMSO) and propidium iodide (PI) 2200× (stock 20 mM in DMSO).

### Live/dead experiments

For microscopy evaluation, black 96 well microtiter plates (89621, Ibidi) were used. Two wells with each bacterium were inoculated with 300 μl. A single position was chosen in each well and a 5 × 5 tile scan of z-stacks of 10 sections with 1 µm between each section, field size = 512 × 512 pixels, speed = 9, and averaging = 1.

Imaris was used to estimate the total number of live (green) and dead (red) at different time points (0 h, 2 h, 4 h, 6 h and 8 h). The first 100 volume numbers were confirmed manually to validate Imaris volume estimates. Total number of live and dead PMNs were estimated as a fraction of the total estimated number of PMNs for each time point. The graphs are based on three experiments.

In Imaris the following parameters were used for both aggregates and single-cell bacteria.

**Table:** 

Live PMNs (green):	Dead PMNs (red)
Smoothing: Yes Surface Detail: 0.200 µm Thresholding: Absolute Intensity: 2200 Diameter of largest Sphere which fits into the Object: 3.95 µm Seed Points Diameter: 5.27 µm No upper Threshold	Smoothing: Yes Surface Detail: 0.200 µm Thresholding: Absolute Intensity: 800 Diameter of largest Sphere which fits into the Object: 3.95 µm Seed Points Diameter: 5.27 µm No upper Threshold

### Size restriction experiments

For microscopy evaluation, black 96 well microtiter plates (89621, Ibidi) were used. The experiments were initiated by first adding the bacterial aggregates, defining positions/areas containing different sizes of aggregates, and finally, the addition of PMNs. Aggregate size measurements were done in 2D (biggest range). The bacteria and PMNs were stained with the nucleic acid stains SYTO9 (5 mM, S34934, ThermoFisher) for live cells and PI (0.2 μl/ml of 20 mM in DMSO, Sigma P4170-500 mg) for dead cells. The combination of SYTO9 and illumination from the lasers can increase the phototoxicity of the PMNs [22]. The increase in production of ROS inside the PMNs will kill them and it looks like lysis. Therefore, the imaging was done manually every 20 min.

The plate was placed into the microscope and the lasers were adjusted, 3–5 positions/well were defined of the different sizes of aggregates. Phagocytosis was followed/observed by either time-series imaging or manual observation. Images were taken at 512 × 512 or 1024 × 1024 resolution. Averaging: 1, Zoom:1, Speed: 6–7. The bacteria/PMN interaction was observed manually for up to 4 h. To detect phagocytic PMNs we visualized the PMN’s membrane and the uptake of the bacteria, we used a combination of fluorescence and light microscopy. The size of the ingested aggregates was measured in each file using Zen software and plotted in GraphPad Prism 7.04.

### Microscopic evaluation of live experiments

For microscopic evaluation, the microscope was allowed to warm up 3 h before use. 300 µl of the stained culture (OD_450nm_ = 0.1) was applied to 96 well microtiter plate, uncoated and sterile (Ibidi, Germany) and 100 µl of PMNs were added to give a final concentration of 1 × 10^6^ PMNs/ml. Microtiter plate wells were imaged in an incubation chamber at 37 °C, 5% CO_2_ with a Zeiss LSM 880 confocal laser scanning microscope and the accompanying software Zeiss Zen 2010 v. 6.0. (Zeiss, Germany). Images were obtained with a 63 × oil objective. Image scanning was carried out with 488 nm (green) and 561 nm (red) laser lines from an Argon and DPSS 561–10 laser. Image analysis was performed using the software Imaris v8.3.1 (Bitplane, Switzerland).

### Size measurement of paraffin-embedded tissue samples

A 4-mm punch biopsy was obtained from the axilla of healthy patients, fixed in formalin, embedded in paraffin, sectioned, deparaffinized and stained with PNA-FISH (UniBac) and DAPI (DNA) as in Ring et al. [23]. The size measurement of the aggregates (biggest range) was determined with the measuring tool in the microscope image analysis software Zeiss Zen 2010 (version 6.0; Zeiss, Germany). The graph was generated in GraphPad Prism 7.04.

### Statistical analysis

To determine the effects of bacterial species and aggregation on PMN viability, a linear mixed-effects regression model was fitted with the *lme* package [24]. The logit transformed proportion of viable PMNs was used as the response with time (0-8 hr), aggregation (planktonic or aggregated), and species (PA, SA, EC, SE) as predictors, including all interaction effects as well as a random effect for biological replicate. The *emmeans* package [25] was used to perform pairwise comparisons of (logit transformed) mean proportions of viable PMNS at each time point between species and aggregate status. Tukey *p *value adjustment was utilized to control for multiple comparisons.

To determine the effects of aggregate size on phagocytosis by one PMN, a generalized linear model was fitted to the binary phagocytosis outcome (success or no success) with a logit link function. Aggregate diameter and species were used as predictors in the model, including interactions. To calculate the fold-change effects of aggregate size and species, the coefficients reported by the regression model were back-transformed to probabilities. Predictions of phagocytosis outcome for the three species were performed by generating 100 numbers from 0 to 40um for each species and using the *predict* function to estimate the outcome, as predicted by the regression model.

A Wilcoxon’s rank-sum test was used to compare the size of aggregates attacked by one or more than one PMN.

All statistical tests were conducted at 95% confidence unless otherwise noted. These analyses were performed with R v3.6.0. Plots were generated either in R, utilizing the ggplot2 package [26] or with GraphPad Prism 7.04.

### Results

### Larger aggregates are less likely to be phagocytosed by a single PMN

**Fig. 1:**
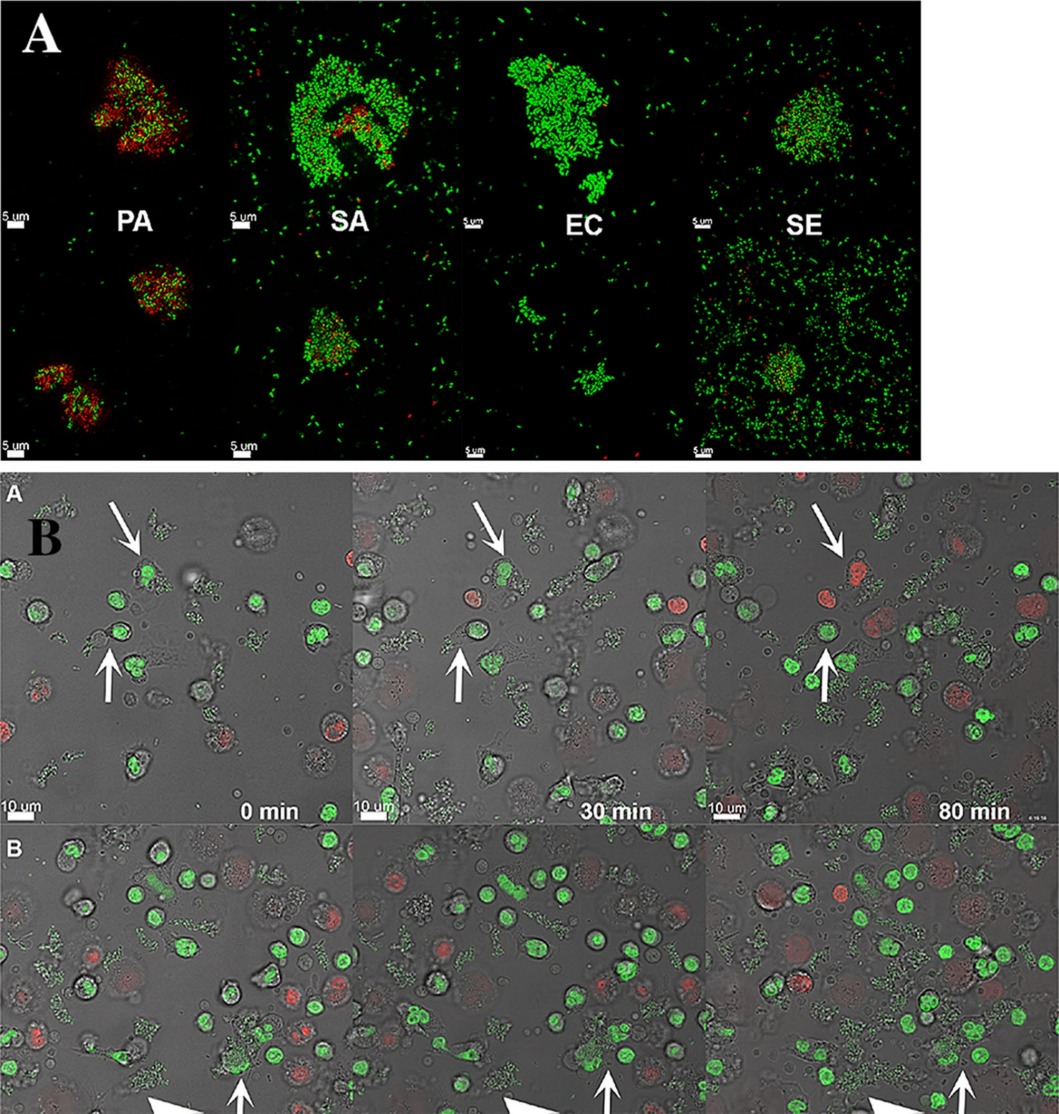
**a** Large and small aggregates of the bacterial strains. Top row: Large aggregates. Lower row: Small aggrerates. *P. aeruginosa* (PA)*, S. aureus* (SA)*, E. coli* (EC) and *S. epidermidis* (SE). The bacterial DNA was stained with SYTO9 for live cells (green) and PI for dead cells (red). **b** Phagocytosis of aggregates by PMNs. Top row shows the phagocytosis of aggregates by one PMN (arrows). One of the PMNs died after 80 min. Bottom row shows phagocytosis by PMNs of a bigger aggregate (arrow) and the big arrow-head points to the growth of aggregates over time. The bacteria are *E. coli.* The bacterial and PMN DNA were stained with SYTO9 for live cells (green) and PI for dead cells (red). Images were obtained every 20 min

**Fig. 2:**
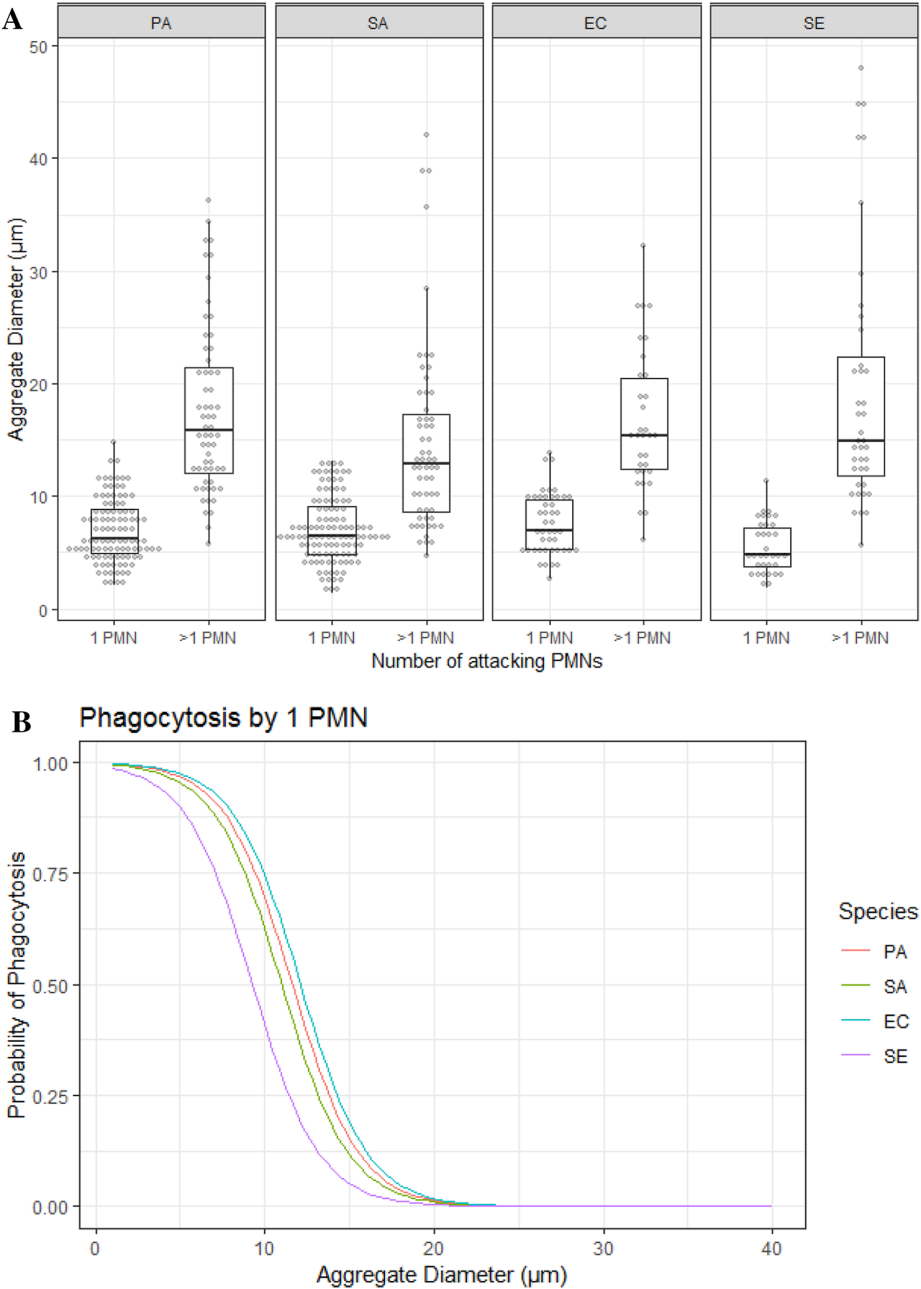
**a** Aggregate size vs phagocytosis by one or more PMNs. Dotplot representing sizes of phagocytosed aggregates for all bacterial species. For each species, the aggregates were divided into those attacked by a single PMN or > 1 PMN. Aggregates are binned by size into bins of 0.7 um. **b** The probability of an aggregate to be phagocytosed. Predicted probability of phagocytosis for aggregates of *P. aeruginosa* (PA)*, S. aureus* (SA)*, E. coli* (EC) and *S. epidermidis* (SE) when attacked by one PMN, based on logistic regression

To investigate whether aggregate size influences the phagocytosis by a single PMN, we examined the outcome (phagocytized vs. not phagocytized) for aggregates of the four bacterial species when attacked by a single PMN. *P. aeruginosa ΔrhlA* and *S. aureus ΔspA* knockout mutants were substituted for the wild-type strains to prevent the bacterial killing of PMNs and allow the bacteria to be phagocytosed. Figure 1a illustrates small and large aggregates of the four bacterial strains. Figure 1b illustrates phagocytosis of a small aggregate by one PMN and phagocytosis of a larger aggregate by multiple PMNs. Phagocytosis by one or more PMNs is plotted against aggregate size in Fig. 2a. Mean aggregate sizes for the four strains are presented in Table 1.

**Table 1:** Mean values of aggregate size for the four bacteria from Fig. 3a

	Single PMNs (µm)	Multiple PMNs (µm)
PA	6.9	17.8
SA	6.8	15.2
EC	7.9	16.7
SE	5.6	19.3

The mean diameter of aggregates encountered by one PMN and those successfully phagocytosed for the four bacterial species as well as predictions of phagocytotic success are displayed in Table 2. As expected, there was a significant effect of aggregate size on phagocytosis outcome and larger aggregates were less likely to be phagocytosed (*p* < 0.0001). In general, the probability of being phagocytosed by a single PMN decreased by 1.7-fold for every μm increase in aggregate diameter. Only aggregates of approx. 5 μm diameter were consistently phagocytosed (Fig. 2b). An aggregate of 15 μm had a low chance (between 5 and 18%) of being phagocytosed by a single PMN. The bacterial species also affected phagocytosis and the probability that an aggregate would be successfully phagocytosed was significantly less for *S. epidermidis* than for an equally sized aggregate of the other species (*p* = 0.005).

**Table 2:** Mean aggregate size for all aggregates of each bacterial species

Species	Mean aggregate diamater (µm; ± SEM)		Predicted phagytosis sucess probability		
	Overall	Sucessfully Phagocytosed	5 µm	10 µm	15 µm
PA-rhlA	10.8 (± 2.2)	6.88 (± 0.9)	0.96	0.69	0.15
EC	11.4 (± 3.6)	7.93 (± 1.7)	0.97	0.74	0.18
SA-spA	9.65 (± 3.8)	7.04 (± 1.1)	0.95	0.62	0.11
SE	13.1 (± 6.3)	5.48 (± 1.3)	0.89	0.40	0.05

### Aggregate phagocytosis by multiple PMNs

To investigate how to aggregate size affects clearance by multiple PMNs, we observed aggregates attacked by multiple PMNs during the phagocytosis experiments. The mean size of aggregates attacked by multiple PMNs is displayed in Table 3. As expected, the mean diameter of aggregates encountered by multiple PMNs was significantly larger than those attacked by single PMNs (*p* < 0.001, Fig. 2a). For the PA-*rhlA* mutant, we observed that multiple PMNs could phagocytose aggregates with sizes up to approximately 37 μm. The mutant did not appear to kill the PMNs and we observed degradation of aggregates after phagocytosis in some PMNs, while others moved out of the frame of view. For the SA-*spA* mutant, some PMNs died while attempting to phagocytose the aggregate, while others survived. New PMNs did not appear to be attracted to the aggregate. Additionally, some PMNs died after phagocytosing pieces of the aggregate. For *S. epidermidis*, multiple PMNs engulfed larger pieces of the aggregate and then moved on. *S. epidermidis* did not appear to be virulent to the PMNs. For *E. coli*, similar to the other species, multiple PMNs attacked larger aggregates and degraded smaller pieces of the aggregate before moving away, which allowed additional PMNs to attack the aggregate (Table 4).

**Table 3:** Descriptive Statistics for aggregates attacked and phagocytosed by > 1 PMN

Species	Diameter		
	Mean	SEM	Max
PA	17.6	2.3	36.31
SA	14.8	3.6	42.05
EC	16.7	3.2	32.25
SE	19.5	6.3	47.97

**Table 4:** Bacterial strains used in this study

Strain	Relevant characteristic	Source and/or reference
*Pseudomonas aeruginosa* (PA)	Wild type	University of Washington, Seattle, WA, USA.
*Pseudomonas aeruginosa* (PA-*rhlA)*	rhlA::Gm_r_, containing plasmid…	University of Washington, Seattle, WA, USA. (Morten Alhede et al 2009)
*Staphylococcus aureus* 8325-4 (SA)	Wild type	Hanne Ingmer (University of Copenhagen; Copenhagen, Denmark)
*Staphylococcus aureus* 8325-4 *spA* (SA-*spA*)	*Dspa::tet5*	Hanne Ingmer (University of Copenhagen; Copenhagen, Denmark)
*Escherichia coli* 042 (EC)	Wild type	Karen Krogsfelt (State Serum Institute, Copenhagen, Denmark
*Staphylococcus epidermidis* ATCC 14990 (SE)	Wild type	Hanne Ingmer (University of Copenhagen; Copenhagen, Denmark)

### Bacterial species and aggregation affect killing of PMNs

**Fig. 3:**
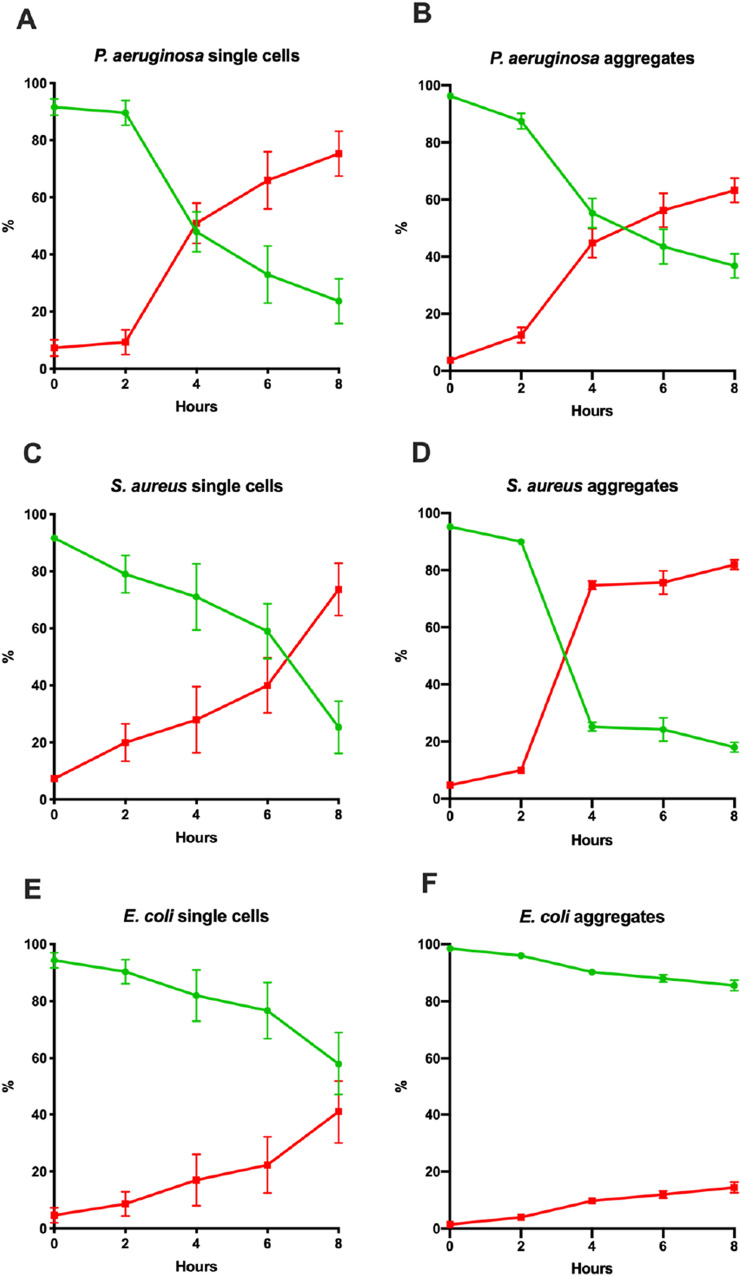

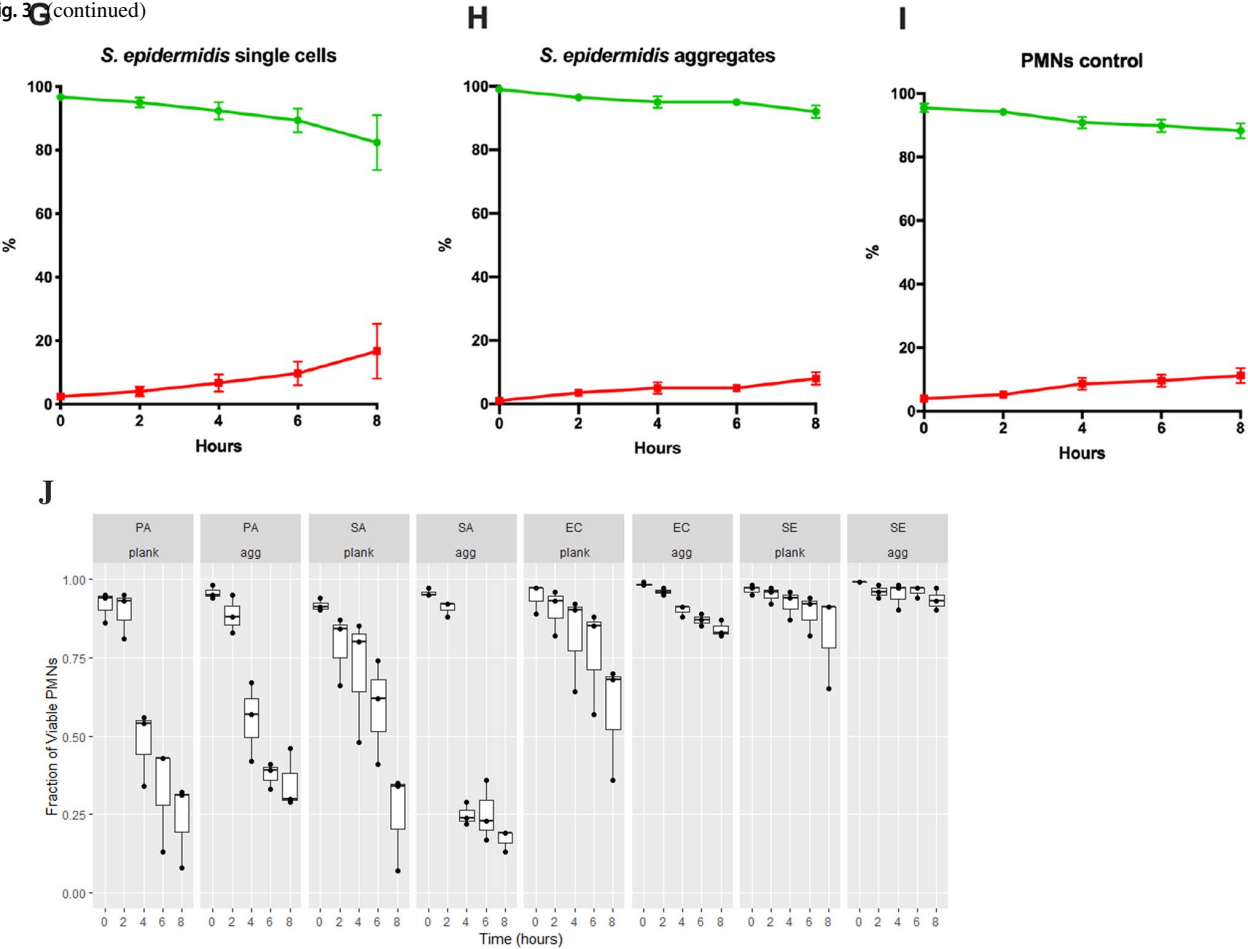
**a**–**i** Killing of PMNs by the four bacteria when as single cells or aggregates. Single-cell bacteria or aggregated bacteria were inoculated with PMNs at 0 h. At different time points a 2D image was obtained and the ratio of live (green) and dead (red) PMNs were estimated using Imaris software. **a**, **b** *P. aeruginosa*, **c**, **d** *S. aureus*, **e**, **f** *E. coli*, **g**, **h** *S. epidermidis* and i control PMNs. Each point represents the mean of 3–4 experiments together with SEM. j Proportion of viable PMNs compared to total number of PMNs following incubation with single cells or aggregates of *P. aeruginosa* (PA), *S. aureus* (SA), *E. coli* (EC) and *S. epidermidis* (SE) from 0, 2, 4 and 8 h post-inoculation (*n* = 3). Box-plots represent the median and inter-quantile range (IQR), while the whiskers represent 1.5*IQR

Since we observed rapid killing of PMNs by wild-type *P. aeruginosa* and *S. aureus,* we estimated the killing of PMNs by all four, wild-type bacterial species, and established the rate of killing when bacteria were either as single cells or aggregates. Single or aggregated cells were inoculated with PMNs and inspected microscopically at 0 h, 2 h, 4 h, 6 h and 8 h (Fig. 3a–i). The PMNs were stained with SYTO9 (green) and propidium iodide (PI, red), which stain the DNA of viable and non-viable cells, respectively. The proportion of viable PMNs for each condition is displayed in Fig. 3j. Incubation with single cells of all species significantly reduced the proportion of viable PMNs. There was a time-dependence of PMN killing for all species and significant reductions in the proportion of viable PMNs only became apparent at 4 h post-inoculation (*p* < 0.05). Single cells of *S. epidermidis* had the smallest effect on PMN viability and killed 18% ± 9% (mean ± SEM) after 8 h. *E. coli* had killed 42% ± 11% after 8 h. *P. aeruginosa* had killed 52% ± 7% of the PMNs after 4 h and 76% ± 8% after 8 h. *S. aureus* killed 41% ± 9% of the PMNs after 6 h and 75% ± 9% after 8 h. At 8 h, *P. aeruginosa* and *S. aureus* had killed a significantly higher proportion of PMNs than *S. epidermidis* or *E. coli*. The effect of aggregation of the bacteria on PMN killing was significant, but species dependent. Only *S. aureus* demonstrated significantly higher reduction of PMN viability when aggregated, compared to single cells (*p* < 0.05). PMN controls showed a reduction in viability of 5–8% over 8 h in the absence of bacteria.

### Aggregate size of skin aggregates in healthy individuals

**Fig. 4:**
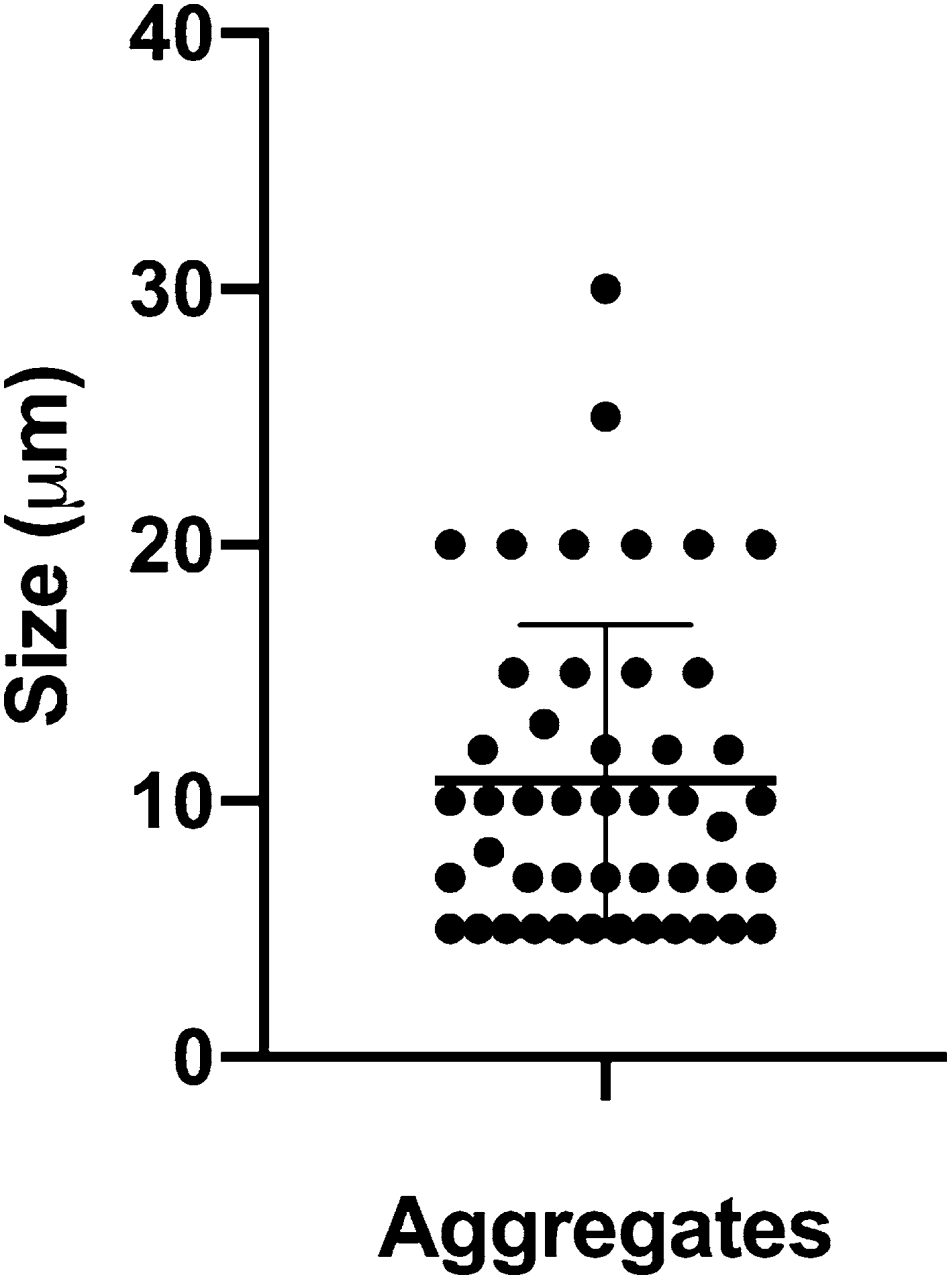
Aggregate size of skin aggregates in healthy individuals. Tissue sections from a 4-mm punch biopsy at the axilla, were deparaffinized and stained with PNA-FISH (UniBac) and DAPI (DNA). Tissue sections were obtained from 7 healthy patients. The graph shows the measurement of 47 aggregates from 1to 6 sections from each patient together with the SD. The measurements were performed using the measuring tool in the microscope image analysis software Zeiss Zen 2010

It has previously been shown that the bacterial aggregates size found on the stratum corneum of healthy patients on average is between 10 and 50 μm [23]. Here we show that in the moist stratum corneum aggregates are on average approx. 10 μm. Figure 4 represents measurements of aggregates from 7 healthy patients with a mean value of 10.8 μm (Fig. 4). Referring back to our data from Table 2, this size reflects a 40–75% chance of being phagocytosed, depending on the bacterial species.

### Discussion

This study demonstrates associations between bacterial aggregate size and phagocytosis by PMNs. Overall, our results indicate that larger bacterial aggregates are less likely to be phagocytosed by PMNs. The probability of successful phagocytosis, in general, decreased 1.7-fold for every μm increase in aggregate diameter. Only aggregates of approx. 5 μm in diameter were consistently phagocytosed by a single PMN. Furthermore, the aggregate size of naturally occurring skin microbiota resembles the overall mean aggregate size with only a moderate chance of being phagocytosed by one PMN in vitro.

This study suggests a possible mechanism for the bacterial evasion of host defenses and antibiotics to establish a chronic infection. It is known that, when bacteria succeed in aggregating and forming a biofilm within the host, an infection may become chronic if the aggregated bacteria tolerate antibiotic treatments and phagocytosis. Predisposing risk factors such as immune deficiencies, insertion of implants, or adaptations of the infecting bacteria may render the immune response suboptimal allowing long-term survival and leading to chronic infection [27]. A better understanding of such interactions between bacteria and the human body’s first line of defense may allow interventions that counteract and prevent chronic infection. In this study, we demonstrate that the bacterial species, strain and the aggregation may influence PMN viability. *P. aeruginosa* and *S. aureus* were most potent at killing the PMNs. For the opportunistic pathogen *P. aeruginosa*, rhamnolipid has been shown to be an important factor for the killing of PMNs by inducing lysis of PMNs [18]. *S. aureus* protein A (SpA) has been shown to inhibit phagocytosis [28] and the production of cytolytic toxins such as phenole soluble modulins (PSMs) [19], bi-component leukocidins and hemolysins induce osmotic lysis of the PMNs after being phagocytosed [29]. Furthermore, it has been shown that *S. aureus* is able to induce a form of necrotic cell death of PMNs which results in the release of live *S. aureus* [30] and thereby establishment of a persistent infection [31]. An efficient strategy utilized by bacteria to overcome killing by PMNs is to prevent phagocytosis with physical barriers, such as polysaccharide or polyglutamate capsules. Uropathogenic *E. coli* strains utilize the membrane antigens O75 and K5 to increase the resistance to phagocytosis [32] and antigen 43 for survival inside PMNs after phagocytosis [33]. *S. epidermidis* immune evasion mechanisms are limited to those involving molecules that protect against or eliminate antimicrobial agents secreted by white blood cells [34]. Cheung et al. [34] have shown that *S. epidermidis* has the capacity to produce a toxin with great potential to destroy white blood cells, but its production is at a very limited level [34].

Interestingly, large aggregates of *S. aureus *resulted in increased killing of PMNs which may be due to the selective release of neutrophil extracellular traps (NETs) in response to large pathogens [4]. However, while the aggregate size significantly affected the outcome of phagocytosis for all 4 investigated species, the selective killing of PMNs in response to aggregate size was not observed for *E. coli*, *P. aeruginosa* or *S. epidermidis*. This suggests that our findings cannot be explained by the mode of PMN death, which awaits further studies for identification.

It has been shown that mature, in vitro* S. aureus* biofilms are more difficult for PMNs to phagocytose [35, 36]. Contrarily, in vitro studies of staphylococcal biofilms demonstrate activation of neutrophils after contact with biofilms and phagocytosis in the absence of opsonizing antibodies or complement [37, 38]. Meyle et al. [39] demonstrated that adherence of PMNs to staphylococcal biofilms did not require an opsonization of the biofilm with human serum. Opsonization resulted only in a moderate enhancement of phagocytosis. Additionally, Stroh et al. [38] describe that neutrophils identify *S. aureus* biofilms without the help of opsonizing IgG and complement C3. These data suggested that PMNs alone can recognize and be activated by biofilm components [37].

Interestingly, *S. epidermidis* did not show the increased killing of PMNs when aggregated. *S. epidermidis* biofilms have been shown to be dependent on a extracellular polymer known as polysaccharide intercellular adhesin (PIA) [40], which contributes to resistance of PMN phagocytosis [41, 42]. Another molecule shown to protect *S. epidermis* biofilms from phagocytosis is poly-γ-DL-glutamic acid (PGA) [43].

Neutrophil sensing of microbial size is recognized to selectively release NETs [4]. In addition to the extracellular events of the release of NETs we have found that the aggregate size affects the intracellular accumulation of bacterial pathogens in PMNs, which constitutes a novel explanation for the evasion of aggregates from PMNs.

We compared our in vitro findings to in vivo aggregate sizes and a threshold of tolerance of bacterial aggregates of approx. 10 µm aggregates seems to be maintained in healthy skin by the influence of PMNs. We have previously shown that the skin microbiome is present as widely scattered small aggregates or single scattered cells in the stratum corneum in dry and moist skin [12, 23] and only when external factors affect the skin environment, substantial bacterial aggregates establish [12]. For example, four days of bandage application induce the establishment of medium-sized bacterial aggregates, while the introduction of an acute wound promotes the establishment of significant larger bacterial aggregates at wound edges [12]. Such biofilm aggregate might be the initiation of a chronic wound in susceptible patients. The observed presence of 10 µm bacterial aggregates may be a threshold for pathogenicity. This potential correlation was recently supported by a study of chronic wounds from hidradenitis suppurativa (HS) patients using PNA-FISH and CLSM. The authors found that large bacterial aggregates were significantly associated with a higher presence of inflammatory cells (lymphocytes, neutrophils and macrophages) [7].

Previously, the sizes of in vivo biofilms from different chronic infections have been measured to be between 4 and 1200 μm, where the biggest are implant-associated ranging from 500 to 1200 μm. Most aggregates in soft tissue infections ranged from 5 to 200 μm [9]. Such large bacterial biofilm is only established when opportunistic conditions occur, promoting the proliferation of the microbiota and overwhelming the threshold of tolerance.

The microbiome is comprised of an enormous number of bacteria that reside in and on the body with a symbiotic relationship with their human host [44]. The bacteria from the microbiome were previously considered contaminants when detected in implant-related chronic infections, but a recent review indicates that, often, bacteria of our own microbiome is responsible for these infections [45]. Accordingly, chronic infections can be caused by bacteria (*S. epidermidis*, *Propionibacterium acnes* and *S. aureus* for nasal carriers), which are generally considered harmless but become pathogens after breaching the immune barrier.

Most studies examining phagocytosis use macrophages which, despite resembling neutrophils, are larger in size and contain granules of anti-microbial agents. These studies examine phagocytosis of different sizes of spherical beads, but do not indicate what happens to the neutrophil or macrophage—do they degrade the bead? Do they burst? What happens if the bead is too large to phagocytose? For macrophages, it has been shown that shape, not size, of the object impacts phagocytosis. Size has an impact on the outcome of phagocytosis when the particle volume exceeds the macrophage volume [46]. Mechanisms of neutrophil phagocytosis have been investigated by analyzing one-on-one encounters between neutrophils and microbes using suction to fixate the neutrophil at the tip of a glass pipette and the microbe at another [47]. This method demonstrated that beads of 11 um are not phagocytosed by a neutrophil [48]. However, such a one-on-one encounter likely does not occur in vivo and the co-operation of the neutrophils to degrade a large aggregate was not examined. There is also a downside to the method used in this study since we measured the sizes of the aggregates by the longest axis, rather than in three dimensions. Consequently, we cannot say how many bacterial cells were included or how thick/deep the phagocytosed aggregates were.

In this study, we intended to elucidate whether a size restriction of aggregates exists for phagocytosis and investigate any ultrastructural evidence on how it affects the PMNs.

We hypothesize that, if the aggregates become too large, circulating PMNs may not be able to phagocytose them quickly enough, which may lead to chronic infection. PMNs must be recruited before an aggregate becomes too large. Large aggregates e.g. due to altered skin microbiome of the patient or slow/reduced PMN recruitment may explain the 1–2% of infections that become chronic.
